# Advanced AI Techniques for Dementia Prediction using MRI Imaging

**DOI:** 10.1002/alz70856_107381

**Published:** 2026-01-10

**Authors:** Saira Kiran, Jingchun Chen

**Affiliations:** ^1^ University of Nevada Las Vegas, Las Vegas, NV, USA; ^2^ UNLV‐NIPM, Las Vegas, NV, USA

## Abstract

**Background:**

Dementia is a broad category of cognitive decline that affects a person's ability to perform everyday tasks. Unfortunately, there is no cure for dementia, and diagnosis usually happens at later stages when symptoms have significantly progressed. Early detection and accurate staging of dementia could slow down symptom progression and improve the quality of life for affected individuals. In recent years, deep Learning algorithms have emerged as a powerful tool in the early dementia diagnosis. By utilizing MRI images, deep learning techniques can effectively classify dementia at different stages, enabling quicker and more targeted interventions.

**Method:**

We used an MRI dataset from Kaggle, categorized into four classes: non‐dementia, very mild dementia, mild dementia, and moderate dementia. The dataset was highly imbalanced, with only 1% of images representing moderate dementia. To address this, we trained a 6‐layered Convolutional Neural Network (CNN) using two approaches: (1) a standard 6‐layer CNN model and (2) a CNN with SMOTE (Synthetic Minority Over‐sampling Technique) to mitigate class imbalance. Model performance was evaluated using accuracy, precision, recall, F1 score, confusion matrix, as well as accuracy and loss curves.

**Result:**

Our SMOTE‐CNN model, incorporated the SMOTE technique, showed significant improvements in classifying the four categories of dementia compared to the standard CNN model. The SMOTE‐CNN model achieved an overall accuracy of 99%, while the standard CNN model had an accuracy of 71%. Precision, recall, and F1‐scores for the SMOTE‐CNN model were also notably higher, with perfect scores (1.00) for the 'Mild‐Demented' and 'Moderate‐Demented' classes, and near‐perfect scores (0.99) for the 'Non‐Demented' and 'Very Mild‐Demented' classes. These results highlight the effectiveness of SMOTE in addressing class imbalance and improving model performance for dementia stage classification.

**Conclusion:**

The results highlight deep learning's potential in enhancing dementia prediction and early detection, leading to better disease staging and management. By addressing class imbalance, we significantly boosted model performance. Future work should focus on validating with independent datasets and incorporating more balanced data to improve generalizability. Additionally, applying the CNN model to real‐world data could further enhance dementia prediction and public health outcomes.

